# Latent Genetic Effects of Past Selection on Blood Feeding: History Matters

**DOI:** 10.3390/insects13100939

**Published:** 2022-10-16

**Authors:** William E. Bradshaw, Piper Kizziar, Rudyard J. Borowczak, Ethan Kirsch, Christina M. Holzapfel

**Affiliations:** Institute of Ecology and Evolution, University of Oregon, Eugene, OR 97403-5289, USA

**Keywords:** *Wyeomyia smithii*, mosquito, biting behavior, genetic correlation, latent immunity

## Abstract

**Simple Summary:**

Conventional wisdom argues that strong directional selection over time decreases genetic variation, thus reducing adaptive ability in an ever-changing world. We find the opposite. Directional selection on blood feeding in low-biting, southern pitcher-plant mosquitoes results in an avid biting population. When selection is withdrawn and no longer imposed, biting rapidly reverts to the original, ancestral state. When selection is re-imposed on the previously selected population, side-by-side with the unselected control population, biting quickly resumes in the selected line, but, importantly, not in the control line, contrary to expectations. Hence, past selection leaves an enduring legacy on a population. History matters.

**Abstract:**

Conventional wisdom is that selection decreases genetic variation in populations, variation that should enable and be essential for population persistence in an ever-changing world. Basically, we find the opposite. Response to selection on biting in the pitcher-plant mosquito, *Wyeomyia smithii*, increases from 20 to 80% in 19 generations, but reverts back to the original 20% after seven generations of relaxed (not reversed) selection. At the same time, biting in the control line remains at the original 20% through 30 generations without blood feeding. Imposition of selection on biting in both lines elicits a rapid response in the previously selected line, but, importantly, not in the control line. Genetic variation for biting has increased, not decreased, as a consequence of long-term directional selection, contrary to expectations. Convergent phenotypes belie the underlying difference in future adaptive potential. Selection events over time in the background of individuals or populations will determine outcomes of applied research, be it in the fields of medicine, agriculture, or conservation. In short, history matters.

## 1. Introduction

Darwinian evolution is based on three observations that are widely accepted: (1) organisms vary, (2) organisms tend to increase geometrically, and (3) not all organisms survive. Darwin’s insight was that during the struggle for existence, some organisms perish, while others survive and reproduce, and in so doing, pass on to their offspring the basis for their success [1] (pp. 31–33). In essence, evolution is any change in gene frequency in a population of organisms from one generation to the next, regardless of whether that change is the result of random sampling of the genome (genetic drift) or the result of selection. “Artificial” selection occurs when the experimenter determines which individuals in a population reproduce based on a specific trait or combination of traits; “natural” selection occurs when the environment determines which individuals in a population reproduce, regardless of the traits involved. Both forms of selection can take place in either the laboratory or in nature.

Dobzhansky and Spassky [2] imposed directional selection for positive or negative geotaxis (artificial selection) in laboratory populations of *Drosophila*. Following 20 generations of response to this artificial selection, selection was terminated and flies were maintained in the laboratory without selection (relaxed selection) for 20 additional generations, during which time natural selection resulted in flies reverting towards, but not converging with the original geotactic “score” 40 generations previously. They did not explore response to renewed directional selection after relaxed selection.

Application of an organophosphate to control *Culex pipiens* imposes artificial selection on resistance alleles at an *esterase* locus. Mosquitoes with the resistant alleles realize lower fitness in the absence of the organophosphate. A geographic gradient of decreasing pesticide application results in a corresponding cline of decreasing resistance alleles at the *esterase* locus [3,4]. In this case, pesticide imposes tradeoffs between artificial selection and ongoing natural selection and, as artificial selection is relaxed, natural selection rises in importance, analogously to Dobzhansky and Spassky’s *Drosophila* [2].

Regardless of the agent of selection, response to that selection depends upon genetic variation underlying the trait or traits being selected [5] (Equation (11.2)): R = h^2^S, where response to selection (R) is the product of the heritability (h^2^), a measure of the “extent to which phenotypes are determined by genes transmitted from parents” [5] (p. 113), times the strength of selection (S) applied to that trait.

In accord with John Donne, (Donne J (1623) Meditation XVII. “No man is an island, entire of itself; every man is a piece of the continent, a part of the main.): No *gene* is an island, entire of itself; every *gene* is a piece of the *genome*, a part of the *whole organism*. After selection on geotaxis, Dobzhansky & Spassky [2] (p. 78) also cited “slight but significant” correlated responses in body size, eye size, testis color, and wing venation, which also “showed some indications of instability [reversion] after relaxation of selection.” Such correlated responses to selection result from an underlying genetic correlation between the two traits. Hence, if directional selection is applied to a trait, whether in the laboratory or in nature, and there is a correlated response to that selection, then there must be (1) heritable variation in the first trait, (2) heritable variation in the correlated trait, and (3) a genetic correlation between the two traits (Box 1).

Box 1 **Correlated response to selection [5] (Equation (19.6)).**

$CR_{Y}=\frac{S_{X}}{\delta_{PX}}\left(h_{X}h_{Y}r_{A}\delta_{PY}\right)$

CR_Y_ = correlated response in trait Y to direct selection on trait X

S_X_ = selection differential imposed on trait X

δ_PX_ = phenotypic standard deviation in trait X

δ_YX_ = phenotypic standard deviation in trait Y

h_X_ = (heritability of trait X)^1/2^

h_Y_ = (heritability of trait Y)^1/2^

r_A_ = additive genetic correlation between trait X and trait Y

For there to be a correlated response, each of the elements in the parentheses must be non-zero.

In effect, response to strong directional selection on a trait becomes the major determinant of fitness, overriding other, but relatively minor correlated determinants of fitness, imposing a “cost of correlation” with respect to fitness [6,7]. When selection is relaxed, then natural selection again comes into play, overriding previous artificial selection, and the selected trait reverts towards pre-selection values.

Herein, we confirm and elaborate on this general pattern in the pitcher-plant mosquito, *Wyeomyia smithii* (Coq.) (Diptera: Culicidae). We show that positive selection for blood feeding in a polymorphic population, results in a four-fold increase in propensity to take a blood meal (bite); but, after relaxation of selection, biting rapidly reverts to the unselected (control) phenotype. We interpret this reversion as a cost of blood feeding, as previously shown by an anticipatory commitment to up-regulation of multiple metabolic pathways in biters compared to more opportunistic non-biters [8]. Importantly, in *Wy. smithii*, despite convergence of phenotypes, the formerly selected line harbors greater genetic variation for biting than the unselected control and responds rapidly to renewed selection. History matters.

**Figure 1:**
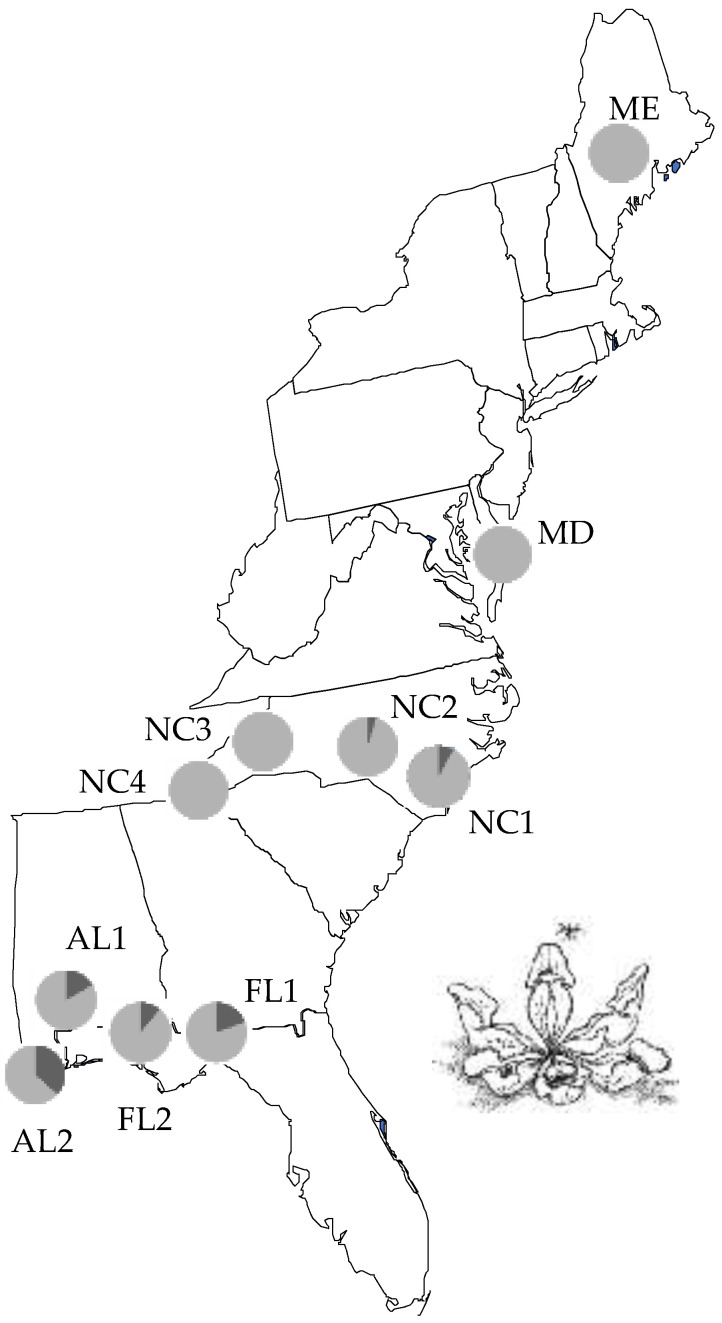
Incidence of biting by *Wy. smithii* in eastern United States during the first generation in the laboratory. The FL, AL, & NC1-2 populations are part of a southern clade; NC3-4 (mountains), MD, & ME belong to the northern clade [12]. Dark gray, incidence of biters; light gray, incidence of non-biters. Selection was imposed on FL1. Plotted from data in Table S1.

The pitcher-plant mosquito, *Wy. smithii* deposits its eggs and completes its entire pre-adult development only within the water-filled leaves of the carnivorous purple pitcher plant, *Sarracenia purpurea* L [9,10]. The range of the mosquito follows that of its host plant from the Gulf of Mexico to northern Canada [11,12]. Northern and mountain populations are obligate non-biting and mature multiple egg batches without a blood meal; southern populations produce the first batch of eggs without a blood meal (autogenous) but require a blood meal (bite) for the second and subsequent ovarian cycles (anautogenous) [13,14]. However, not all southern females bite, with propensity to bite being moderate along the Gulf Coast and low in the Carolina Coastal Plain (Figure 1). The current study is based on over 14,000 larvae collected near Wilma, FL (30° N, 85° W, 10 m elevation).

## 2. Materials and Methods

### 2.1. Standard Rearing Protocol

Selected and control populations were maintained as diapausing larvae on an 8:16 light:dark cycle at 21 °C. To continue generations, populations were transferred to and reared on programmed conditions of a typical summer day at 40° N: an 18:6 light:dark cycle using a sinewave thermoperiod with a maximum temperature of 32 °C and minimum temperature of 15 °C that lagged the light cycle by 3 h. Relative humidity was programmed for a constant 80%. Details and sources in Text S1.

### 2.2. Histogram Method

When hatch of a given generation in the selected or control line exceeded a desired population size, the line was thinned or experimental animals removed using a “histogram” method to minimize unintentional selection on development time in any line. Neither any larvae removed for thinning or for experiments or their progeny were ever returned to the selected or to the control line. Details in Text S1.

### 2.3. Blood Feeding

To determine incidence of biting, ≥390 individuals were reared to adults as above. The number of eclosing females was scored by sexing pupal exuviae (pupal cuticle). Starting at first female eclosion, adults were offered a rat anesthetized with a ketamine/xylazine cocktail for 15 min three times per week between 1200–1400 subjective time (25–30 °C). Any female that engorged blood was scored and recorded as a biter, removed from the cage, and discarded. When females aggregated on the host, any female with a bent labium was scored and recorded as a biting individual and was removed from the cage. The incidence of biting was then calculated by dividing the number of biting females by the total number of females having emerged as adults.

### 2.4. Selection Generations 1–19, Rat Host

**Figure 2:**
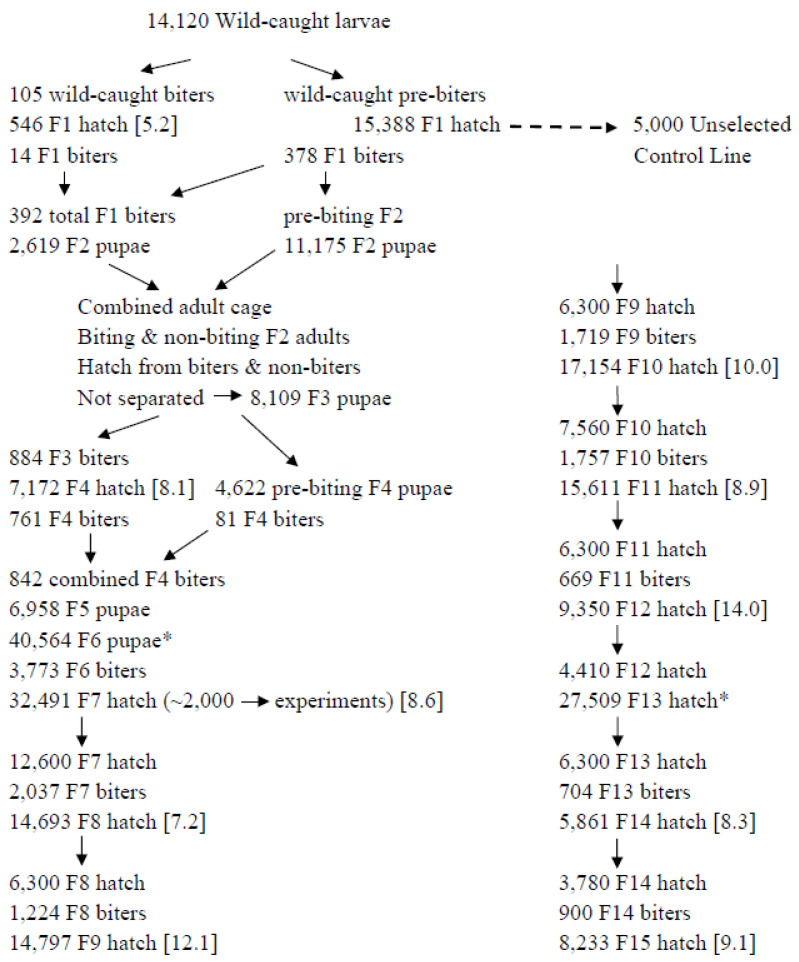
Generation and maintenance of the line selected for avid biting and the unselected control line. The complex manipulations in early generations are due to the fact that, initially, total hatch from biting females was not sufficient to generate a cohort replacement rate (Ro) greater than 1.0. Consequently, until Ro ≥ 1.0 was reached, hatch from biting females was comingled with hatch from females of the same generation that had not taken a blood meal (pre-biters). Until Ro ≥ 1.0, all hatch from blood feeding females were used to generate the selected line; thereafter, hatch in excess of Ro > l.0 were available for experiments. *Reared through one generation without a blood meal to augment the selected line. Hatch from pre-biters represent hatch from eggs produced during the first ovarian cycle, obligately produced without biting. [hatch per biting female].

Selection for biting began using ~14,000 individuals collected from a low-biting population in northern Florida (population WI in other papers from this lab: 31° N, 85° W, 10 m elevation). The environment and protocols used for selection were as above except biters were allowed to feed to completion, removed from their cage, and placed into a separate “biting” cage with supplemental males from the same generation of the selected line. All hatch from the biting cage were used to generate the subsequent generations (Figure 2). Initially, hatch from biting females were not sufficient to maintain a line able to replace itself exclusively from biting individuals. In this situation, we used the abundant hatch generated by females from the same generation, but before they bit (pre-biters), in order to augment the selected line. The selected line was maintained at 10,000 individuals. Note that for each generation of selection after the first, both the pre-biters and the biters were offspring of biters from the previous generation. This protocol was followed until the selected line could sustain itself (R_0_ > 1.0) exclusively from biting females in the 7th generation of selection. Thereafter, the selected line was maintained at 5000 individuals; hatch in excess of 5000 were used in experiments. Through all generations of selection, including those that were not offered a host, hatch were placed on short days (L:D = 10:14) at 21 °C to synchronize each generation and to mitigate inadvertent direct selection on development time, generation time, or the timing of reproductive allocation. After adults of a given generation had died, their offspring were transferred to long days and reared to adulthood, as above.

### 2.5. Response to Selection

Response to selection was determined in the initial P1 = generation 0, and generations 7, 9, 11, and 14, using a rat for a host as described above. Selection was maintained through the 19th generation. After generation 19, both the selected and control lines were maintained separately at 5000 hatch per generation from pre-biters without access to a host. Each generation continued to be synchronized in diapause and individuals in excess of 5000 thinned by the histogram method.

### 2.6. Artificial Host

**Figure 3:**
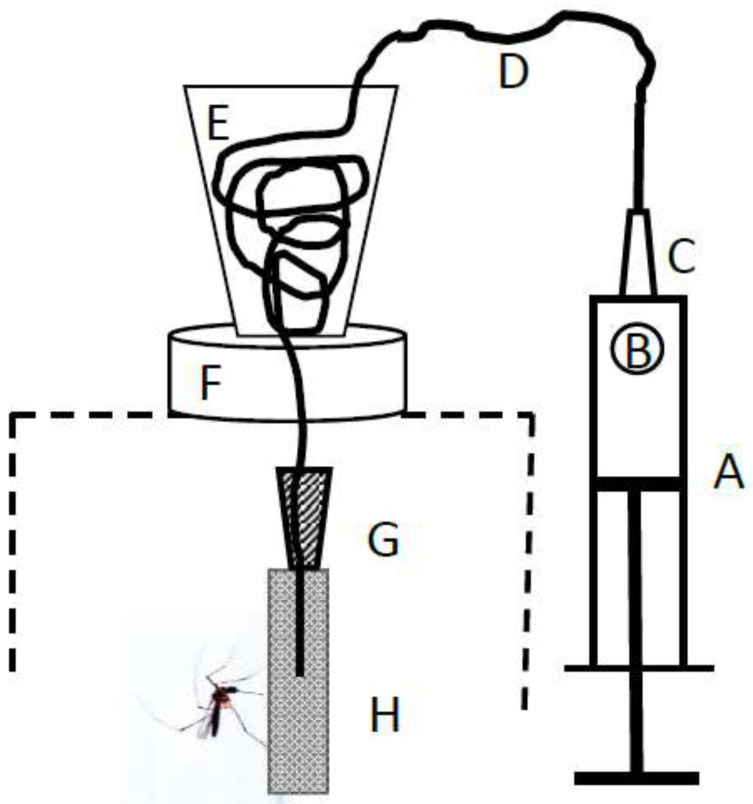
Apparatus for infusing warm CO_2_ through blood-soaked cotton pledgets. A. 50 mL syringe; B. 1–2 chunks of dry ice ca. 1 cm diameter; C. 20 gauge 1.5″ = 3.8 cm needle inserted into D. 1.67 mm diameter poly tubing. E. Poly tubing coiled inside 45 mL cup; F. Foam insulator ca. 1.5 cm thick, 4 cm diameter; G. 18 gauge, 1.5″ = 3.8 cm needle with the end of the poly tubing glued into it; H. cotton roll dental pledget soaked in warm defibrinated sheep blood. Dashed line, top of mosquito cage. Details in Text S1.

To meet the university requirement to obviate the need for live animals for invasive experimental research, we developed an artificial “host” comprised of a heated CO_2_-infused dental pledget soaked in defibrinated sheep blood (Figure 3). To test the efficacy of the artificial host, we compared biting propensity in the biting and the control lines of *Wy. smithii* in the 27th generation with biting by the local tree-hole mosquito, *Aedes sierrensis*, known to be an avid biter in the field and in the lab on a vertebrate host.

Preliminary observations indicated that the blood-soaked pledgets were as successful as a live rat at stimulating blood feeding when cup water temperature was set at 51 °C for *Ae. sierrensis* and 45 °C for *Wy. smithii*. Consequently, cup temperatures of 45 °C were used for all subsequent experiments with *Wy. smithii*.

### 2.7. Secondary Selection

Starting in the 30th generation, the previously selected line and the un-selected control line were exposed to the artificial host as in selection from generations 0–19. Blood-fed females were removed to a separate cage and continued to be offered a host until all biting females had died. Incidence of biting was determined each generation for four generations (30–33). In each generation, the hatch from all blood-fed females were synchronized in diapause and used to found subsequent generations of selection without the addition of any pre-biters.

### 2.8. Statistical Methods

Regression, correlation, and ANOVA used the Excel Analysis Toolpak in Microsoft Office 10. ANCOVA used *JMP Start Statistics* [15]. Standard errors of percentages were estimated as: SE = sqrt(pq/n), where *n* = number of eclosing females, *p* = % biting, q = % not biting, subject to *x*n > 6.0, where *x* is the lesser *frequency* [16] (pp. 134–135).

## 3. Results

**Figure 4:**
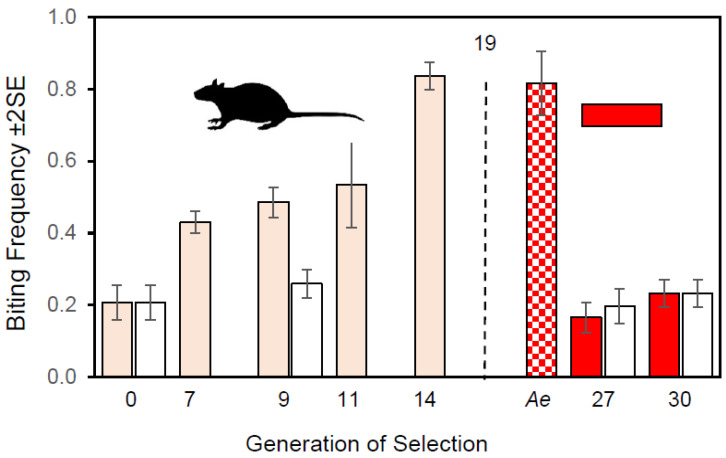
Biting history. Frequency of biting on a rat in the original selected line (pink), the nonbiting control line (white), and on pledgets soaked in defibrinated sheep blood (red). Dashed vertical line marks end of blood feeding on a rat and the start of maintenance of the selected line from non-biters (pre-biters). Frequencies to the right of the dashed line plot biting in generation 27 from blood-soaked cotton pledgets in a viciously biting *Aedes sierrensis* (*Ae*), in the previously selected line (red), and in the unselected control line (white). Error bars represent ± 2 standard errors. Plotted from data in Table S2.

Before selection, 18% of emerging females bit (Figure 4). With increasing selection, biting increased to 84% in generation 14 of the selected line. Hatch per biting female on the rat host (Figure 2) averaged 9.6 ± 0.71SE and was not significantly correlated with generation of selection (r^2^ = 0.11, *n* = 9, *p* = 0.80). Hence, any increase in productivity in the selected line was due more to an increasing incidence of biting than to the resulting fecundity of biters.

Selection continued through the 19th generation. Selection was relaxed after the 19th generation and both the selected and control lines maintained without access to a host until the 27th generation. After relaxed selection, biting in the selected line had converged with the unselected control line in the 27th generation; this result was confirmed in the 30th generation. By comparison with ~20% biting by *Wy. smithii* in the 27th generation, biting by *Ae. sierrensis* exceeded 80% (Figure 4), demonstrating the efficacy of the blood-soaked pledgets in stimulating biting in an avid biting mosquito.

**Figure 5:**
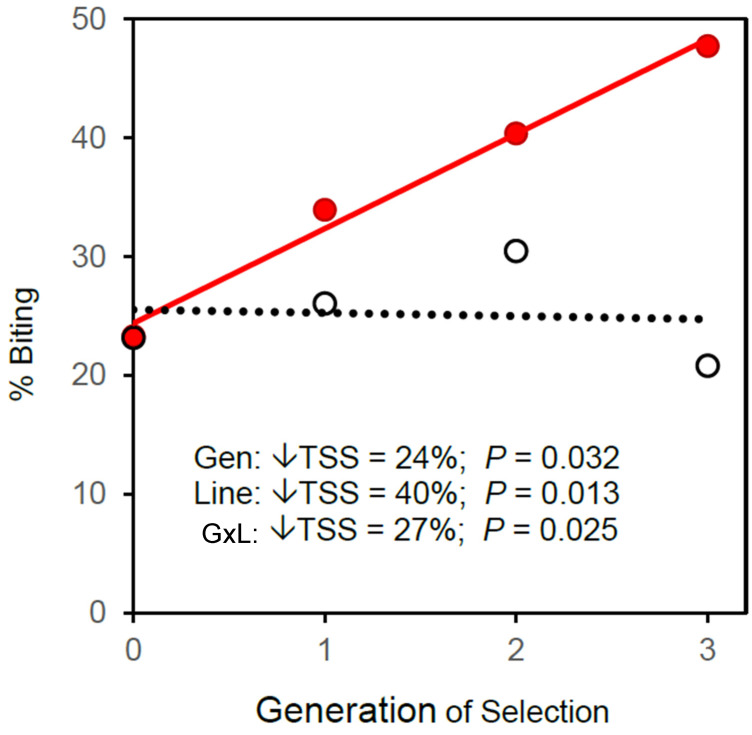
Response to renewed selection for biting starting in Generation 30 on both the previously selected line (solid red symbols & red solid line) and the previously unselected control line (open black symbols & black dotted line). ↓TSS = percent reduction in total sum of squares from ANCOVA (Table S3).

When selection for biting was imposed both on the previously selected line and on the unselected control line, only the previously selected line responded to that selection (Figure 5).

## 4. Discussion

### 4.1. Response to Selection: Generations 0–19

The objective of selection on biting was to develop a biting line to enable comparative genomics between biting and non-biting lifestyles. Selection on biting was successful (Figure 2), demonstrating heritable variation for biting in the initial wild-caught larvae, and generating biting individuals for experiments in the F7 generation. Low early response to selection demonstrated first the necessity of having and maintaining a large population for selection and second, the power of having females produce a first, large batch of eggs before biting (pre-biters). The importance of the latter is that, after the first generation of selection, an increasing proportion of pre-biters were themselves offspring of biters, thereby amplifying the selection process.

### 4.2. Host Shift, Generations 27 & 30

Since the host was switched from a live rat to a blood-soaked pledget, the question arises as to whether the decline in biting in the selected line after blood meals were no longer offered was due to an actual genetic decline in propensity to bite, or to the exposure to a novel host. Two factors indicate that the low incidence of biting by the selected line was due to relaxed selection and not host specificity. First, the high incidence of biting in *Ae. sierrensis* exceeded 80% on the blood-soaked pledget (Figure 4), demonstrating that the artificial host was not inhibiting a naturally avid-biting mosquito. Second, the incidence of biting in the unselected line did not decline below the ancestral level in either the 27th or 30th generation (Figure 4), demonstrating that access to a pledget, in the absence of a rat, did not elicit a lower propensity to bite. We conclude that the initial response to directional selection resulted in an increase from 20 to 80% biting females due to artificial selection and that the decline back to 20% in generations 27 and 30 was due to relaxed selection followed by natural selection, and not to exposure to a novel host.

### 4.3. Costs of Correlation

Cohort replacement from blood-fed females exceeded 1.0 in the seventh generation of selection, enabling us to use the excess for experiments without compromising ongoing selection on blood feeding in a large line. From this excess, 2000 individual *Wy. smithii* were used to compare gene expression between biting and control lines [8]. This comparison showed that relative to reluctant biters in the control line, avid biters in the selected line upregulated proteosomal, spliceosomal, ribosomal, odorant receptor, and RNA and DNA synthesis genes. The commitment to up-regulated metabolic pathways were made before a blood meal was actually consumed and whether that blood meal actually took place or not. Hence, up-regulation of these pathways represents a “cost of correlation” in response to directional selection on biting in *Wy. smithii*.

Southern populations of *Wy. smithii* are polymorphic for biting (Figure 1) while northern populations are obligate non-biting and produce multiple batches of eggs without a blood meal [13,14,17,18]. The question is then whether the evolutionary transition from blood-feeding (anautogenous) to obligate non-biting (autogenous) in nature occurred as a result of isolation and drift or was due to selection during the northward range expansion of *Wy. smithii*. A total of 1459 genes are differentially expressed between the control line and the line directly and specifically selected for blood feeding [8] (Figure 2). Of those 1459 genes, 95% of them were the same genes that varied in the same direction between the selected biters and an obligate non-biting northern population. Up-regulated pathways in response to artificial selection for biting were the same pathways downregulated by natural selection during the evolutionary divergence of obligate non-biting from facultatively biting populations. Therefore, the metabolic costs of correlation revealed by artificial selection in the laboratory were specifically those costs mitigated by natural selection in nature.

### 4.4. Latent Effects of Blood Feeding

Propensity to bite persists at the baseline level in the unselected control through 30 generations without access to a host or source of blood (Figure 4). At the same time, after eight generations of relaxed selection, propensity to bite in the selected line converges with the unselected control and remains stable at this value through three additional generations. In combination, this persistence, convergence, and stability indicates that a low level of genetic propensity to bite is being maintained in the Florida population by some form of balancing selection, unrelated to blood feeding, per se.

Despite phenotypic convergence of the selected with the control line in generations 27 and 30 (Figure 4), renewed response to selection (Figure 5) showed that the selected line harbors greater genetic variation for propensity to bite than the control line. Phenotypic convergence has not resulted in genetic convergence. The latent effects of prior selection persist in the selected line and remain available for future evolutionary adaptation. History matters.

## 5. Conclusions

Contrary to conventional wisdom, our results show that directional selection can and does increase variation in the fundamental genetic architecture of individuals and populations. This change is clearly illustrated in *Wy. smithii* having undergone 19 generations of directional selection followed by a period of relaxed selection, and then renewed directional selection. Despite phenotypic convergence with the control line, the previously selected line harbors latent genetic variation that enhances future evolutionary potential. Therefore, the selection history of the individuals or population with which one is working will determine the success of any management practice employed, be it in the field of medicine, agriculture, or conservation.

## Data Availability

Supporting data are available in the Supplementary Materials.

## Supplementary Materials

The following are available online at https://www.mdpi.com/article/10.3390/insects13100939/s1, Table S1: Geographic variation in propensity to bite for Figure 1; Table S2: Biting history for Figure 4 and Figure 5; Table S3: ANCOVA for Figure 5; Text S1: Materials & methods details.
